# Increased double strand breaks in diabetic β-cells with a p21 response that limits apoptosis

**DOI:** 10.1038/s41598-019-54554-8

**Published:** 2019-12-18

**Authors:** Vanessa S. Y. Tay, Surabhi Devaraj, Tracy Koh, Guo Ke, Karen C. Crasta, Yusuf Ali

**Affiliations:** 1 Lee Kong Chian School of Medicine, Nanyang Technological University, Singapore, Singapore; 2 School of Biological Sciences, Nanyang Technological University, Singapore, Singapore

**Keywords:** Cell death, Senescence

## Abstract

DNA damage and DNA damage response (DDR) pathways in β-cells have received little attention especially in the context of type-2 diabetes. We postulate that *p21* plays a key role in DDR by preventing apoptosis, associated through its overexpression triggered by DNA stand breaks (DSBs). Our results show that β-cells from chronic diabetic mice had a greater extent of DSBs as compared to their non-diabetic counterparts. Comet assays and nuclear presence of γH2AX and 53bp1 revealed increased DNA DSBs in 16 weeks old (wo) db/db β-cells as compared to age matched non-diabetic β-cells. Our study of gene expression changes in MIN6 cell line with doxorubicin (Dox) induced DNA damage, showed that the DDR was similar to primary β-cells from diabetic mice. There was significant overexpression of DDR genes, *gadd45a* and *p21* after a 24-hr treatment. Western blot analysis revealed increased cleaved caspase3 over time, suggesting higher frequency of apoptosis due to Dox-induced DNA strand breaks. Inhibition of *p21* by pharmacological inhibitor UC2288 under DNA damage conditions (both in Dox-induced MIN6 cells and older db/db islets) significantly increased the incidence of β-cell apoptosis. Our studies confirmed that while DNA damage, specifically DSBs, induced *p21* overexpression in β-cells and triggered the p53/p21 cellular response, p21 inhibition exacerbated the frequency of apoptosis.

## Introduction

β-cells within the pancreatic Islets of Langerhans provide insulin to maintain glucose homeostasis. Loss and/or dysfunction of β-cells leads to insulin insufficiency and frank type-2 diabetes (T2D)^1^. Adult β-cell numbers are maintained through self-replication rather than through differentiation from a stem-cell progenitor^2^. The frequency of β-cell replication is low, making them vulnerable to stressors such as ER stress, glucolipotoxicity, oxidative stress and/or DNA damage. Each of the above pathways has been shown to contribute to declining β-cell function and mass in T2D^3^. Among these, β-cell DNA damage and DNA damage response (DDR) pathways have received comparatively less attention in the context of T2D^4^. In most eukaryotes, a typical response to DNA damage is cell cycle arrest, which allows for DDR to occur. If the damage is too severe, cells will either activate an apoptotic or a senescence programme^5,6^. There is evidence that the β-cell is no different, as prolonged and severe DNA damage disrupts metabolic homeostasis in diabetes^7,8^. Also, cellular response to DNA damage has been implicated in the impairment of glucose metabolism and loss of pancreatic β-cell mass^9,10^.

The type of DDR depends on both the extent of damage, as well as the type of stressor (e.g. ultraviolet or oxidative damage)^11^. For instance, mild DNA damage induces cell cycle arrest that is reversible thus allowing the cell to repair. However, prolonged DNA damage triggers senescence and/or apoptosis^5^. As a result, cells arrest either at the G_1_/S or the G_2_/M phase of the cell cycle. Additionally, DDR also triggers an inflammatory response in senescent cells. This causes an upregulation of cytokines and adhesion molecules which in turn recruits other inflammatory cells. This persistent inflammation further accelerates the disease progression. The key mediators of DNA damage signalling and response are the tumour suppressor *p53* as well as cyclin dependent kinases (CDKs) *p21* and *p16*^12,13^. Cellular senescence is regulated by the p53/p21 and p16/Rb signalling pathways with *p53* being the most important transcriptional factor involved in the DDR^14–16^. While the former signalling pathway induces cell cycle arrest and senescence, the latter is required for the maintenance of senescence. Senescence once established by the p16/Rb pathway is irreversible^15^.

DNA damage has been implicated in the development of both type-1 diabetes (T1D) and T2D. DNA damage in β-cells is seen to be an early event in T1D, contributing to autoimmunity and exacerbating T1D pathology^17^. DNA damage in T2D is known to be caused by a variety of stimuli. For instance, oxidative stress in T2D patients was responsible for significantly higher DNA damage in lymphocytes leading to a decreased efficiency of DNA repair^9,10^. In another study, glucolipotoxicity due to high fat diet in mice led to cellular senescence in β-cells^18^. Another factor recently implicated in increased DNA damage was congenital hyperinsulinism in patients. In these rare cases, glucokinase mutations were seen to cause DNA double strand breaks (DSBs) in β-cells leading to dysfunction and apoptosis^7^.

While DNA damage is known to be a contributing factor towards T2D pathology, the extent to which DSBs contribute to β-cell dysfunction and death during T2D remains unknown. The finding of increased DSB frequency in β-cells of patients with congenital hyperinsulinemia prompted an investigation into DSB presence in β-cells of diabetic mice (db/db mice). We further probed DDR gene expression in primary β-cells and in MIN6 cells exposed to Dox, to establish the β-cell response to DSBs. Our results show that DSBs are higher in older diabetic (db/db) islet cells compared to those from younger diabetic (db/db) mice. The DDR pathway triggered in these islet cells was seen to be aligned to the p21 response pathway rather than the p16 pathway in our diabetic mouse model of choice. Chemical induction of DSBs using Dox in MIN6 cells revealed a similar mechanism and pharmacological inhibition of *p21* disrupted the DDR process and increased the incidence of β-cell apoptosis. Together, the evidence presented here points to increased DSBs in older db/db mice and that *p21* plays an essential role in DDR and β-cell survival in diabetic β-cells with DSBs.

## Materials and Methods

### Animal studies

All animal procedures and methods were performed in accordance with the protocol and ethical regulations approved by the Institutional Animal Care and Use Committee (IACUC) of the Nanyang Technological University Singapore, Singapore (IACUC 140905/A0373). B6.BKS(D)-Leprdb/J mice were purchased from Jackson Laboratories, USA and nondiabetic control litter mates were used at ages 10 and 16 wo. The mice were maintained on an alternating 12 hr light/dark cycle in temperature controlled rooms and were given free access to food and water. For the STZ experiments, 14–16 wo C57BL/6Inv mice were used (InVivos Pte Ltd, Singapore). Streptozotocin (STZ) (Sigma-Aldrich) was dissolved in citrate buffer immediately prior to injections and was administered intraperitoneally at a concentration of 150 mg/kg. Mice were sacrificed 24 hrs after STZ had been administered.

### Mouse islet isolation

Mice of the required age were euthanized and the bile duct was clamped at the duodenal entry. Collagenase type V (Sigma-Aldrich) (0.8 mg/ml) was perfused into the bile duct. Pancreata was then removed and incubated for 6–9 minutes at 37 °C with gentle agitation. Once digestion was complete, samples were washed with RPMI medium (Gibco) with 10% foetal bovine serum (FBS), 1% penicillin / streptomycin and 25 mM HEPES. Islets were then hand-picked from the digested debris and either dissociated into single cells for comet assay or left to recover overnight in CMRL media prior to treatment and/or RNA isolation.

### Single cell dissociation of islets

Picked islets were dissociated with Accutase^®^ solution (Sigma-Aldrich) for 7 minutes before being mechanically dispersed with gentle pipetting. Dissociated cells were then washed with RPMI medium and number of cells counted before use in Comet Assay Analysis (TriTek).

### Comet assay

Single-cell comet assays were performed according to the manufacturer’s instructions (Trevigen). Islets isolated from wild-type (WT), db/db and STZ treated mice were re-suspended in cold phosphate buffered saline (PBS) at 2 × 10^5^ cells/ml, mixed with low-melt agarose (1:10 ratio) and spread on frosted glass slides. After the agarose solidified, the slides were sequentially placed in lysis and alkaline solutions (Trevigen). Slides were then subjected to electrophoresis at 12 V for 10 min in 1X Tris-borate-EDTA (TBE) buffer, fixed with 70% ethanol, and stained with 4′, 6-diamidino-2-phenylindole (DAPI). Nuclei were visualized using epifluorescent illumination under a 20X objective on a Nikon wide-field microscope and images were analysed with the NIH Image J program. DNA damage was quantified for 100 cells for each experimental condition by determining the percentage of DNA in tail using Comet Score (TriTek) software, using the formula % DNA in tail = (total intensity of tail / total intensity of comet) × 100. Mean comet tail DNA % was expressed as a percentage of the positive control, STZ (mean of three independent experiments).

### Histological processing of animal tissue samples

Mice (10 wo and 16 wo) were euthanized and their pancreata harvested. The collected tissues were fixed in 4% paraformaldehyde (PFA) for 24 hrs followed by 24 hrs in 30% sucrose for cryo-protection. Following which, tissues were embedded in Optimal Cutting Temperature (OCT) medium and kept frozen at −80 °C till further use. Frozen tissue blocks were sectioned at 10 µm using a cryostat (Leica), mounted on SuperFrost Plus ™ adhesive slides (Thermo Scientific) and kept at −80 °C prior to immunofluorescence staining.

### Immunofluorescence staining

Slides were retrieved and air dried prior to staining. After rinsing in 1x Tris buffered saline (TBS), they were blocked with 1x TBS / 1% bovine serum albumin/10% FBS/0.3 M glycine for 1 hr at room temperature. Slides were then stained with anti-phospho-histone γH2AX (Merck), anti-53bp1 (Bethyl Laboratories) and anti-Insulin (Dako) overnight at +4 °C. Next, the slides were rinsed and incubated with secondary antibodies, anti-rabbit Alexa 488, anti-rabbit Alexa 647, anti-mouse Alexa 488, anti-guinea pig Alexa 547 (Life Technologies) and DAPI for 1 hour at room temperature. Finally, slides were rinsed in 1x TBS before being mounted with Hardset Antifade mounting medium (Vectashield) and a glass coverslip.

### Confocal imaging and image analysis

Immunofluorescent stained slides were imaged with the LSM800 inverted confocal microscope with Airyscan (Carl Zeiss). All images were digitally acquired at 40x magnification. Islets (2–5) were selected randomly per animal and the number of positively stained cells were manually counted and segregated into 3 categories: cells that contained nuclei with less than 5 phospho-histone γH2AX foci, more than 5 but less than 20 and those with more than 20 positive foci.

### Immunoblot analysis

MIN6 cells were seeded in 24-well plates at a density of 3.5 × 10^5^ cells per well and grown for 24 hrs before treatment with 500 ng/ml Dox (Sigma-Aldrich, adriamycin) for a time course of 10, 30, 120 minutes; 6, 16 and 24 hours. After incubation, cells were rinsed, lysed with radioimmunoprecipitation assay (RIPA) buffer (Sigma-Aldrich) containing protease and phosphatase inhibitors. Protein extracts were then separated using the Novex NuPAGE MES-SDS gel, following which they were transferred to a nitrocellulose or Polyvinylidene fluoride (PVDF) membrane for immunoblotting. The blots were incubated in blocking buffer followed by β-actin (Abcam) for 1 hr at room temperature or caspase-3 (Cell Signalling), cleaved caspase-3 (Cell Signalling) or p21 (Abcam) overnight at +4 °C. Next, the blots were incubated with anti-mouse and anti-rabbit horseradish peroxidase (HRP), and visualised using ECL Plus (Cell Signalling) and Gel Doc imager and Image Lab (Bio-Rad Laboratories).

### Cell culture

MIN6 cells were cultured in high-glucose Dulbecco’s Modified Eagle Medium (DMEM) supplemented with 10% FBS, 1% penicillin / streptomycin, 0.1% β -mercaptoethanol.

### Doxorubicin, UC2288 treatment and qPCR

MIN6 cells as well as isolated mouse islets were treated with either doxorubicin (500 ng/ml; Sigma-Aldrich, Adriamycin) and/or UC 2288 P21 inhibitor (2.5 µM; Abcam, ab146969) for 24 hrs. Cells or islets were then lysed and RNA was extracted using RNeasy Plus Mini kit (Qiagen) and RNeasy Plus Micro kit, respectively. Purified RNA was then converted into cDNA using the high-capacity cDNA reverse transcription kit (Applied Biosystems). cDNA was used for qPCR to quantify the transcript expression level of the genes of interest. The relative levels of mRNA expression were analysed by qPCR using intron-spanning primers (Universal ProbeLibrary, Roche) using the QuantStudio™ 6 Flex Real-Time PCR System (Applied Biosystems).

### Caspase/Apoptosis assay

Caspase 3/7 activity was determined using Apo-ONE(R) Homogeneous Caspase- 3/7 assay kit (Promega) according to the manufacturer’s instructions. Briefly, cells or islets were lysed using RIPA lysis buffer and the amount of protein was quantified using BCA (Bicinchoninic acid) assay method. 20 µg of protein was loaded for each sample in 96 well black plates along with assay buffer and substrate. The fluorescence was measured on a Synergy HTX microplate reader.

### Statistical methods

Data collected was tested for normality (Kolmogorov-Smirnov) and thereafter expressed as mean ± SEM, with a minimum of 3 independent experiments each. Statistical significance was determined using student t-test for comparison between two groups or by ANOVA for multiple groups with p < 0.05 determined to be statistically significant (Graphpad Prism).

## Results

### Widespread DNA strand breaks observed in islets of old diabetic mice

**Figure 1:**
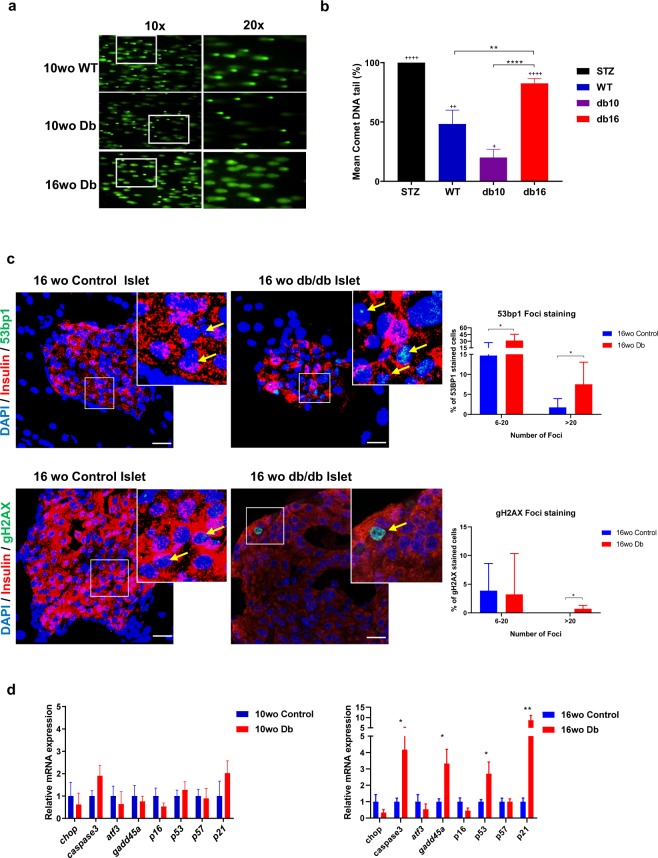
Increased DNA damage was observed in islets from 16 week old db/db mice. (**a**) Comet assay for DNA damage in mice islet cells; 10 weeks old (wo) control islets (extent of damage:++), 10 wo db/db islets (extent of damage:+) 16 wo db/db islets (extent of damage:++++). (**b**) Mean comet DNA tail % (+ = significance compared to STZ). (**c**) Maximum intensity projection images of pancreatic sections stained with 53bp1 and γH2AX, together with insulin and DAPI in 16 wo db/db islets confirmed extensive presence of DNA double-strand breaks and significantly higher % of cells showing more than 20 foci for each DNA strand break marker as compared to 16 wo control, non-diabetic β-cells. Scale bar denotes 20 µm. (**d**) Increased DNA Damage in 16 wo db/db islets was evidenced by the enhanced expression of *p53, p21, caspase3* and *gadd45a* - key players in the DDR pathway – as compared to age matched non-diabetic mice and 10 wo db/db mice. All data in this figure represents mean ± SEM, n = 5 mice. *P*-values were calculated using Student’s t-test *p < 0.05, **p < 0.01, ***p < 0.001 vs control.

DNA damage has been known to occur in β-cells of various diabetic mouse models. However, the nature of damage and cellular response to β-cell DNA damage is relatively unclear. We used comet assay, also known as single-cell gel electrophoresis (SCGE), to measure the extent of DNA damage in islets from old diabetic mice and observed increased DNA damage in older mice. To the best of our knowledge, this is the first time a comet assay has been applied to primary β-cells from both control and diabetic mice. The comet tails are proportional to the degree of DNA damage. We were able to show, that not only were the comet tails significantly longer in 16 wo db/db islets as compared to age-matched control islets, but they were also much longer than 10 wo db/db islets (Fig. 1a). 10 wo db/db islets had lesser DNA damage compared to age-matched control islets which is not surprising given that at 10 weeks, db/db islets are expanding and secreting more insulin to compensate for peripheral insulin resistance. The compensation in such genetically obese animals starts to decline after 12 weeks of age^19^. The percentage of DNA in comet tails of 16 wo db/db islet cells was 86% suggesting that a majority of islet cells during this time are subjected to some form of DNA damage (Fig. 1b). Noteworthy, in rodent islets, and especially so for db/db islets, β-cells make up more than 95% of islets cells. Alkaline comet assays are unable to distinguish between single-strand breaks and double-strand breaks. Hence to determine whether diabetic β-cells suffer from increased DNA double-strand breaks, we stained pancreatic sections of diabetic and control mice with γH2AX, a marker for DNA DSBs^20^. We observed increased insulin^+^ γH2AX^+^ staining in 16 wo db/db mice compared to 10 wo db/db (≤0.5% of β-cells with 6–20 γH2AX foci) as well as non-diabetic mice, suggesting that double-strand breaks do occur at a higher frequency in β-cells of older diabetic mice. Another marker of DNA strand breaks is 53bp1, a protein that is recruited to the site of strand breaks for repair. We observed significantly higher number of insulin^+^ 53bp1^+^ cells in 16 wo db/db islets as compared to control islets which further confirmed extensive DSBs in chronic diabetic islets. The % of cells in 16 wo db/db islets with 20 foci or greater for 53bp1 was much higher when compared with age-matched control islets (Fig. 1c). To analyse the nature of DDR, as a result of DSBs, a qPCR of known DDR genes was carried out in islets from both 10 and 16 wo db/db mice, together with their age-matched non-diabetic controls. 16 wo db/db islets were observed to significantly overexpress DDR genes such as *caspase3, gadd45a, p53* and *p21* when compared to the controls. Changes in these genes were not observed in cDNA from 10 wo db/db islets (Fig. 1d). Taken together, these results suggest that chronic diabetic β-cells are subjected to a greater extent of DNA strand breaks compared to non-diabetic animals and the frequency of strand breaks is higher in older diabetic mice.

### p21 and not p16 is seen to be significantly upregulated in β-cells subjected to DNA strand breaks

**Figure 2:**
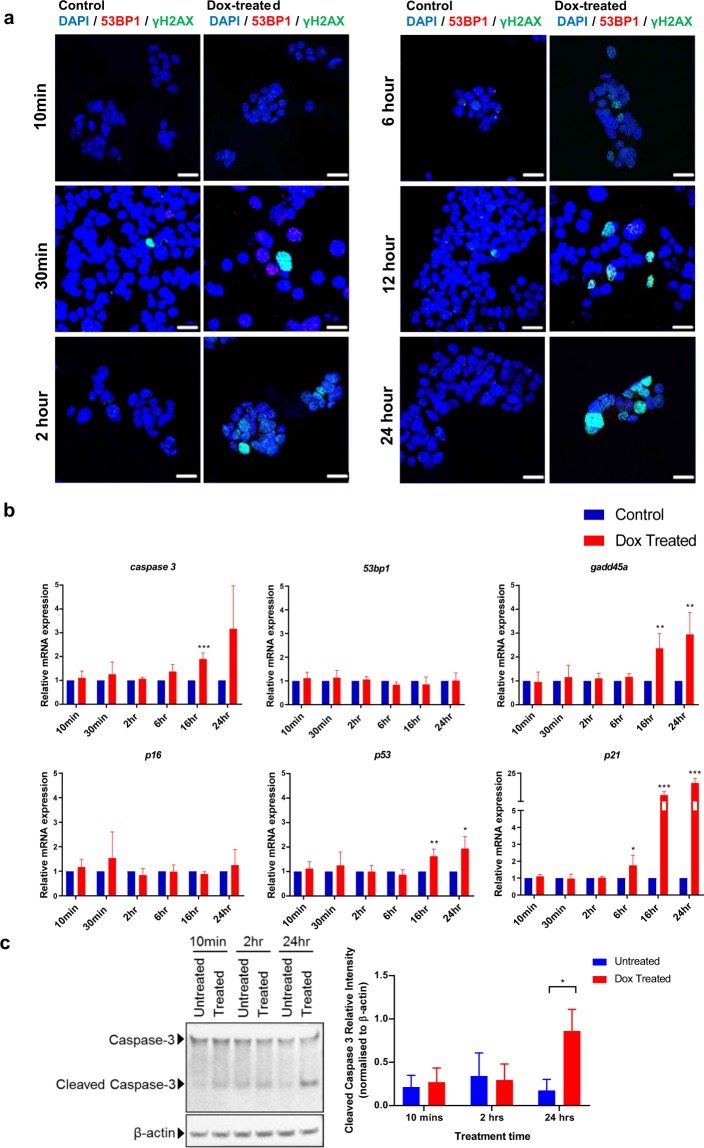
Time course study of doxorubicin induced DNA damage and cellular response in MIN6 cells. (**a**) Immunofluorescence Staining of Min-6 cells with DAPI (blue), 53bp1 (red) and γH2AX (green) after treatment with doxorubicin (Dox) from 10 mins to 24 hrs. Scale bar denotes 20 µm. (**b**) Gene expression kinetics in MIN6 cells treated with Dox. All data in this figure represents mean ± SEM, n = 4. (**c**) Representative immunoblot for caspase3, cleaved caspase3 and the corresponding β-actin (loading control) levels from MIN6 cells treated with Dox. Semi-quantitation of the cleaved caspase 3 band intensities, normalized to β-actin band intensities at the different timepoints post-Dox treatment was done using ImageJ software. Data represents mean ± SEM, n = 3. All *P*-values were calculated using Student’s t-test *p < 0.05, **p < 0.01, ***p < 0.001 vs control.

Increased DNA strand breaks were observed in 16 wo diabetic mice compared to non-diabetic age-matched littermates as well as younger (10 wo) diabetic animals. In response to double-strand breaks, eukaryotic cells mount a DDR which involves the recruitment of DNA repair proteins to the damaged DNA sites. Visualizing DDR using immunofluorescence techniques is challenging because positive foci only show up when more than 100 protein molecules concentrate at a certain site^21^. To circumvent this, we determined gene expression changes of known DDR genes such as *p53, p21, p16, atf3, gadd45a* over time in mouse insulinoma cells (MIN6 cells)^7^. DNA double strand breaks were induced by treating MIN6 cells with Dox for varying time periods from 10 mins to 24 hrs. In order to confirm DNA damage due to Dox (500 ng/ml), MIN6 cells were stained for both γH2AX and 53bp1 after exposure to Dox for differing time periods (10 mins, 2 hrs, 6 hrs, 12 hrs and 24 hrs). Immunofluorescence staining confirmed that Dox-induced DSBs with increasing number of foci for γH2AX from 10 mins to 24 hrs (Fig. 2a). A significant population of the cells showed DNA damage starting from 2 hrs post treatment with Dox, with extensive positive staining for DSB recruiting protein 53bp1 at 24 hrs post-Dox treatment. The gene expression time-course study for Dox-treated MIN6 resembled that of 16 wo db/db islets (Fig. 1d), with significant overexpression of *p21* and other DDR genes, namely, *caspase3*, *gadd45a* and *p53* post 6 hrs of treatment. Expression of *p21* steadily increased from 6 hours with a fifteen-fold increase in expression at 24 hrs suggesting that *p21* is a β-cell response gene that increases in expression with prolonged exposure to DSBs (Fig. 2b). Our data indicates that while *p21* was significantly overexpressed, there was no significant change in the *p16* expression levels. This suggests that the cells did not enter into a senescent state but were marked for DNA repair or apoptosis^22^. As *caspase3* (known mediator of apoptosis) was also seen to increase in expression with Dox treatment (Fig. 2b), we then measured protein levels of both caspase3 and cleaved caspase3 (active form and inducer of apoptosis) at 10 mins, 2 hrs and 24 hrs by western blot. Western blot analysis at the different time-points post-Dox treatment showed increased cleaved caspase3 expression over time, with a significant difference at 24 hrs (Fig. 2c). This suggests increased frequency of apoptosis in β-cells subjected to DNA double strand breaks.

### Inhibition of p21 under DNA damage conditions significantly increases expression of known DDR genes and increases incidence of apoptosis in MIN6 cells

**Figure 3:**
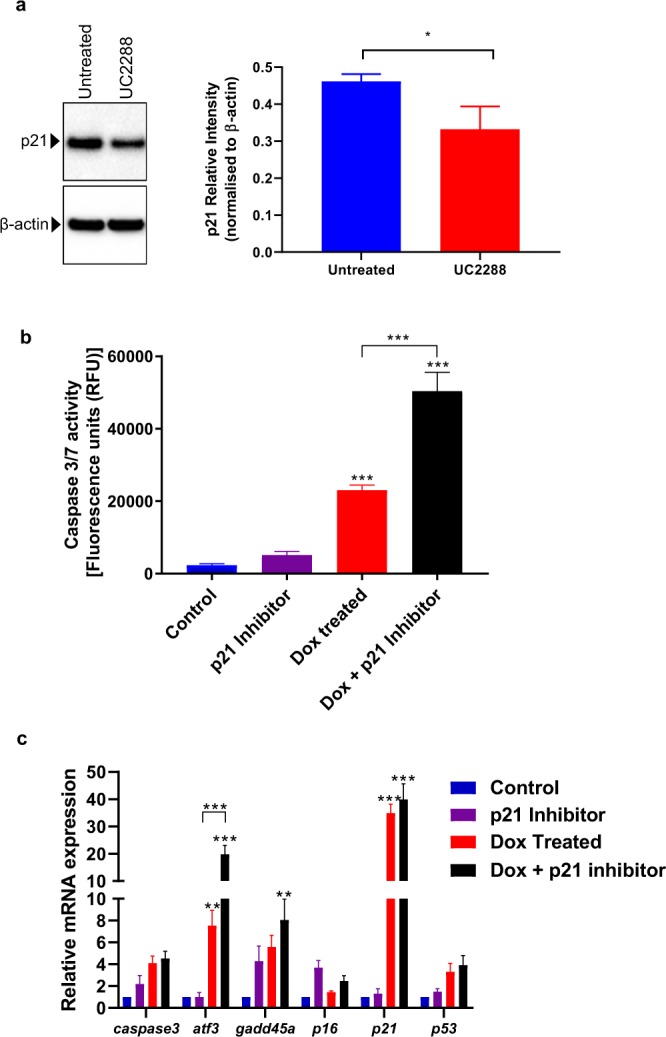
Inhibition of *p21* under DNA damage conditions leads to heightened DDR and increased incidence of apoptosis in MIN6 cells. (**a**) Representative immunoblot of p21 and corresponding β-actin (loading control) levels from MIN6 cells treated with UC2288 (2.5 µM) as well as the semi-quantitation of p21 band intensities normalized to β-actin band intensities using Image J. (**b**) Caspase activity assay shows increased incidence of apoptosis in MIN6 cells treated with p21 inhibitor under DNA damage conditions. (**c**) Addition of *p21* Inhibitor UC2288 along with Dox causes increased expression of DDR genes, *atf3* and *gadd45a* as well as *p21*. All data in this figure represents mean ± SEM. *P*-values were calculated using Student’s t-test for apoptosis (n = 4 experimental replicates) and ANOVA for gene expression (n = 6 experimental replicates); *p < 0.05, **p < 0.01, ***p < 0.001 vs control.

To determine whether the increased expression of *p21* played a role in DSB-induced apoptosis, we inhibited p21 function using a small molecule p21 inhibitor drug, UC2288 (Abcam, ab146969). UC2288 was shown to selectively inhibit p21 at a high dose (10 µM) and was shown to decrease p21 protein levels^23^. In our hands, 10 µM UC2288 led to a very high frequency of MIN6 cell death at 24 hrs (data not shown), prompting us to titrate its concentration down further. At a dose of 2.5 µM, UC2288 significantly reduced p21 protein levels (Fig. 3a) with no significant effect on MIN6 viability after 24 hrs. Using an apoptosis kit, we then measured caspase 3/7 activity in each of the treatment groups to see how UC2288 (2.5 µM) affects apoptosis under Dox-induced DSB conditions. Increased caspase 3/7 activity was observed in MIN6 cells treated with Dox (2.5 µM) for 24 hrs corroborating the increased expression of cleaved caspase3 seen with the western blot (Fig. 3b). Inhibition of p21 under Dox-induced DSB conditions was seen to increase caspase activity by more than two-fold as compared to Dox treatment alone, with no change in caspase activity upon treatment with p21 inhibitor alone. This observation corroborated with an earlier study which showed in a different cell line that p21 inhibited Dox-induced caspase-3 activity and apoptosis^24^. MIN6 cells when treated with both Dox and p21 inhibitor for a period of 24 hrs were found to have a significantly higher expression of the DDR genes, *atf3* and *gadd45a* as compared to cells treated with Dox alone (Fig. 3c). This clearly demonstrated that inhibition of p21 under DSB conditions induced by Dox, heightened the DDR in MIN6 cells. Therefore, we established that while p21 inhibition alone in MIN6 cells did not lead to β-cell apoptosis, its inhibition under DSB conditions increased the frequency of apoptosis in β-cells. This shows that *p21* is essential to the functioning and survival of β-cells under DSB conditions.

### Inhibition of p21 under DNA damage conditions in primary β-cells exacerbates DDR and increases incidence of apoptosis similar to MIN6 cells

**Figure 4:**
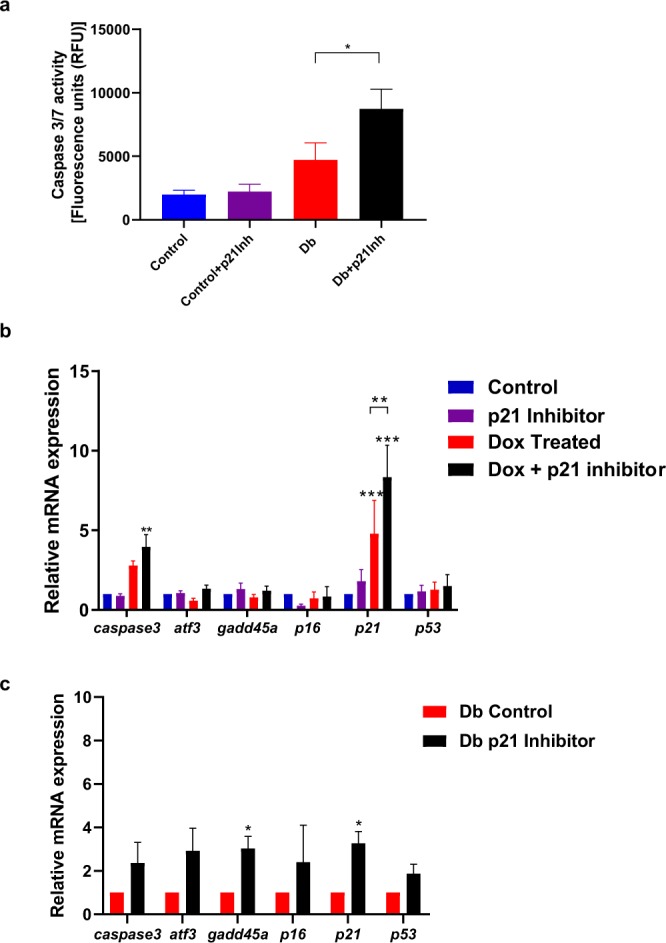
Inhibition of p21 under DNA damage conditions leads to heightened DDR and increased incidence of apoptosis in mouse islets similar to MIN6 cell line. (**a**) Caspase activity assay showed increased apoptosis mediated by caspase activity in 16 wo db/db islets treated with p21 inhibitor under DNA damage conditions (db/db islets already shown to have extensive DNA damage - Fig. 1) when compared with age matched control islets. (**b**) Addition of p21 Inhibitor UC2288 along with Dox caused increased expression of *caspase3* in 16 wo control mouse islets. (**c**) Addition of p21 inhibitor to 16 wo db/db islets showed overexpression of DNA damage response gene, *gadd45a*. All data in this figure represents mean ± SEM. *P*-values were calculated using Student’s t-test (4a and 4c; n = 3) and using ANOVA (4b, n = 6) *p < 0.05, **p < 0.01, ***p < 0.001 vs control.

Our previous results with MIN6 cells suggested that inhibition of p21 under DSB conditions increases the expression of DDR genes and the apoptotic activity in β-cells. As cell lines are known to deviate in their response from primary cells, it was important to verify whether similar DDR gene expression changes occurred in both MIN6 cells and primary β-cells. Islets from 16 wo control and db/db mice were isolated and left to recover from the isolation process overnight. Primary β-cells were subsequently treated with either Dox (500 ng/ml) or p21 inhibitor alone (UC2288, 2.5 µM) or a combination of both for 24 hrs. We have previously established that db/db islets show significantly higher DNA damage as compared to their age matched lean counterparts (Fig. 1). Therefore, db/db islets were treated with only p21 inhibitor for 24 hrs. We first verified the caspase activity levels in 16 wo control as well as db/db islets treated with p21 inhibitor for 24 hours using the Apo-ONE caspase assay. Here, control islets, both with and without the addition of p21 inhibitor were used as controls, with the former serving as a baseline indicator for caspase activity as a result of p21 inhibitor alone. We found that addition of p21 inhibitor alone to the control islets caused no significant change in the caspase activity levels in the absence of DNA damage (Fig. 4a). However, when db/db islets (already established to have DSB) were treated with p21 inhibitor for 24 hrs, there was significantly higher caspase activity suggesting increased frequency of apoptosis (Fig. 4a). We further determined if the same findings regarding expression of DDR genes were replicable in primary β-cells. For control islets, addition of p21 inhibitor under conditions of DSB induced by Dox caused a significant increase in the expression of DDR gens, *caspase3* and *p21* (Fig. 4b) Similarly in db/db islets incubated with p21 inhibitor for 24 hrs, while there was a trend of upregulation of most DDR genes, only *gadd45a* and *p21* showed significant overexpression (Fig. 4c). Noteworthy, *p21* expression was not significantly altered in both MIN6 and 16 wo control islets (modest but insignificant change) exposed to UC2288. Our findings with primary β-cells thus corroborate results from our studies with MIN6 cell lines. Once again, this highlights the importance of p21’s role in amelioration of apoptosis and maintenance of β-cell function under DNA damage conditions.

## Discussion

In this study, we sought to determine whether diabetic β-cells suffered from DSBs to a greater extent compared to non-diabetic β-cells. Knowledge gained here would be key to understanding the contribution of DSBs and β-cell response pathways in cells that are central to the pathogenesis of T2D. Using the alkaline comet assay, we observed significantly higher DSBs in 16 wo db/db islets compared to 10 wo db/db islets, as well as islets from non-diabetic control mice. This suggests that islet cells of older diabetic mice, 90% of which are β-cells, display a greater extent of DSBs. Furthermore, this level was comparable (no significant difference) to islets from our positive control STZ-treated mice. Multiple nuclear γH2AX and 53bp1 foci in insulin-positive cells of 16 wo db/db islets but not 10 wo db/db islets suggests higher DSBs in β-cells of more chronic diabetic mice. Elevated gene expression levels of key mediators of DNA damage signalling, namely *p53, p21*, *caspase3* and *gadd45a*, but not *p16*, from 16 wo db/db islets suggests a p53/p21 pathway response, with *p21* playing a key role in DDR in β-cells. *p21* is of special interest in the context of β-cell dysfunction and/or apoptosis due to the duality of its role (pro and anti-apoptotic) as observed in β-cells as well as other cell types^11,25,26^. DNA damage induces expression of *p53* which in turn mediates the upregulation of *p21*. p21 is a cell cycle inhibitory protein and thus is a key player in the maintenance of β-cell mass. *p21* plays an important role in cellular response to DNA damage by regulating cell cycle progression, transcription, apoptosis and repair^27^. It inhibits proliferation of cells by preventing transition from G_1_/S to the G_2_/M phase of the cell cycle by inhibition of cyclins that are essential for the progression of cell cycle, specifically cdk-2 kinase^25,28,29^. *p21* plays a role in cellular arrest in the G_1_/S phase by inhibiting cyclin E, and cyclin A/CDK2^30^. *p21* may also negatively regulate cell cycle by binding PCNA (Proliferating Cell Nuclear Antigen) thus interfering with DNA synthesis^31^. From our experiments with diabetic mouse islets, it was evident that increased DSBs in primary β-cells corresponded with an increased expression of *p21*. We next performed a time course study *in vitro* using MIN6 cells exposed to Dox, from 10 mins to 24 hrs. MIN6 cells when exposed to Dox, showed increased expression of *p21, p53*, *gadd45a* as well as *caspase3*, but not *p16* similar to our previous results with islets. Elevated levels of *caspase3* expression, corresponded with higher intensities of cleaved caspase3 at 24 hrs. This strongly suggests that the increased incidence of β-cell apoptosis in Dox-treated MIN6 cells was due to DSBs. Thus, in both diabetic mouse islets as well as the MIN6 cell line exposed to Dox, we observed increased *p21* expression along with increased *caspase3* expression as a result of DDR to strand breakages. We used the chemotherapy drug Adriamycin (Dox) as a tool to specifically determine the DDR pathway following extensive DNA strand breaks in MIN6 cells and found that *p21* gene upregulation was mirrored in both diabetic islet cells and MIN6 cells exposed to Dox. Cytokines such as interferon-γ and interleukin-1 have been shown to induce DNA damage in islets through increased NO production^32^, but the nature of this damage is not specific to DSBs^33^. Hence, we chose Dox treatment for its DNA strand break specificity. Noteworthy, a recent cohort study on patients receiving chemotherapy (such as those on Dox) showed that cancer survivors had a subsequent higher risk of developing type-2 diabetes^34^.

*p21* can prevent apoptosis, maintain β-cell mass and participate in DNA repair following DNA damage^35–38^. *p21* is shown to be involved in DNA repair through major DDR pathways such as Nucleotide Excision Repair, Nonhomologous End Joining and Base Excision Repair^39–41^. On the other hand, *p21* induces apoptosis in pancreatic β-cells when subjected to endoplasmic reticulum (ER) and oxidative stress^42^. For instance, in ER stress conditions, *p21* promotes apoptosis due to its suppression by *chop*^11^ or by upregulating pro-apoptotic protein Bax^43,44^. Thus, it would seem that inhibition/knockdown of *p21* would be beneficial to maintain β-cell function and prevent apoptosis. However, in studies on islets isolated from rats, Mihalidou and colleagues observed that suppression of *p21* in ER stress conditions led to a lowering of the apoptotic threshold of cells with *p21* ablation, in turn exacerbating ER stress mediated apoptosis^42,45^. Conversely, in a T1D model, *p21* overexpression proved beneficial for β-cell recovery. In STZ treated mice, *p21* overexpression causes the overexpression of several other transcription factors important for the development of pancreatic progenitor cells. Consequently, mouse β-cells treated with STZ had improved recovery due to the differentiation of pancreatic progenitors into islet β-cells^46^. These studies show that the role of *p21* could be dependent on the DNA damage stimulus as well as its cellular concentration. In our hands, subsequent inhibition of p21 under DNA damage conditions using the p21 inhibitor (UC2288), showed increased incidence of apoptosis by more than two-fold compared to Dox-treated cells alone. This suggests that *p21* overexpression triggered by DSBs was essential to prevent β-cell apoptosis. Islets from 16 wo control (Dox-induced DNA damage) as well as db/db islets with or without the addition of p21 inhibitor, similarly showed increased expression of *caspase3* protein. Increased caspase 3/7 activity in db/db islets treated with p21 inhibitor as compared to control islets treated with p21 inhibitor suggests that *p21* activity is required under β-cell DSB conditions to prevent apoptosis. Treatment of UC2288 did not significantly alter *p21* levels in MIN6 cells and 16 wo control islets. On the contrary, increased *p21* mRNA was observed in 16 wo db/db islets exposed to UC2288. Noteworthy, p21 expression was already higher in 16 wo db/db islets compared to control islets. While we are unable to explain this difference in response to p21 inhibition, our data suggests that the inhibition of p21 function (at the protein level) perhaps led to a futile feedback regulation of increased p21 gene transcript levels to compensate for inhibited p21 in db/db islet cells. It is a futile regulation because despite increased p21 mRNA levels in db/db islets (over and above the heightened levels when compared to control islets), the presence of UC2288 still blocks p21 activity, leading to increased apoptosis. Therefore together, our data suggests that p21 is a key intermediary in DDR and its upregulation during DSB conditions is associated with β-cell survival. By means of the comet assay and DSB protein localisation, we showed that β-cells from chronically diabetic mice had increased DNA damage in terms of DNA DSBs. We also demonstrated that *p21* played a key role in β-cells’ DDR by regulating repair and preventing apoptosis. Our findings clearly showed that inhibition of p21 under DNA damage conditions greatly increased the incidence of apoptosis. It is not known whether DSBs and DDR are key to the pathology of T2D in β-cells. But in this context, our data clearly demonstrates that DSBs and the p21-driven DDR are associated with β-cell apoptosis, at least in rodents. Further understanding of these pathways, and of the key players involved in β-cell DDR, might pave the way for therapeutic avenues that could potentially mitigate β-cell apoptosis and stem the progression of T2D.

## Data Availability

All data pertaining to the figures will be made available upon request. Data repository is in progress and DOI and URL will be provided.
